# Kinetic and physicochemical modeling of *β*-galactosidase from *Rhynchophorus palmarum* larvae

**DOI:** 10.1371/journal.pone.0354469

**Published:** 2026-07-22

**Authors:** Sobamfou Marius Kambiré, Mankambou Jacques Gnanwa, David Boa, N’guessan Eugène Jean Parfait Kouadio, Bonito Aristide Karamoko

**Affiliations:** 1 Laboratory of Environment, Climate, Health, Engineering and Sustainable Development, University of Peleforo GON COULIBALY, Korhogo, Côte d’Ivoire; 2 Laboratory of Agrovalorization, University of Jean Lorougnon Guédé, Daloa, Côte d’Ivoire; 3 Laboratory of Environmental Thermodynamics and Physical Chemistry, University of Nangui Abrogoua, Abidjan, Côte d’Ivoire; 4 Laboratory of Biocatalysis and Bioprocesses, University of Nangui Abrogoua, Abidjan, Côte d’Ivoire; 5 European Membrane Institute, University of Montpellier, Montpellier, France; Universidad Nacional Autonoma de Mexico Centro de Nanociencias y Nanotecnologia, MEXICO

## Abstract

Palm weevil (*Rhynchophorus palmarum* L.) is a significant pest that has been identified as a threat to palm trees in tropical regions. Beyond its agricultural impact, its digestive system represents a promising source of biocatalysts. The present study investigates the catalytic activity of *β*-galactosidase extracted from the digestive juice of *R. palmarum* larvae. *o*-nitrophenyl-*β*-D-galactopyranoside (*o*NPG) was utilized as the substrate in this investigation. The purified enzyme exhibited optimal activity at 330.0 ± 1.2 K and pH = 5.0 ± 0.1, as determined by empirical and mechanistic models. The activation energy (E_a_) was estimated at 56.3 ± 9 kJ mol^-1^ using mechanistic models. Furthermore, the pK values for the enzyme-substrate complex were determined to be 4.0 ± 0.1 for the nucleophile and 6.2 ± 0.2 for the proton donor, which provides insight into the catalytic residues. Kinetic analysis through nonlinear regression yielded a catalytic constant (k_cat_) of 4.9 × 10^3^ s^-1^ with V_max_ and K_m_ values of 49 ± 2 U mg^-1^ and 0.77 ± 0.08 mM, respectively. The results obtained provide novel insights into the physicochemical properties of this enzyme. The findings of this study demonstrated that the insect digestive system is a promising and largely untapped source of robust *β*-galactosidases with considerable potential for industrial biocatalytic applications.

## 1. Introduction

Enzymes play a fundamental role in biological systems by catalyzing biochemical reactions with high efficiency and specificity. Among them, β-galactosidases are widely studied due to their involvement in carbohydrate metabolism, particularly with regard to the cleavage of *β*-glycosidic bonds in substrates such as lactose [1]. These enzymes are produced by microorganisms, plants, and animals (including insects) [2–4], reflecting their ubiquity in nature. *β-*galactosidase deficiency in mammals, especially in humans, causes many digestive disorders [5]. Despite the extensive literature on microbial and plant *β*-galactosidases, those derived from insects have received relatively little attention, particularly with regard to their kinetic and physicochemical properties [6,7]. Current research has mainly focused either on identifying new sources of *β*-galactosidases or on improving the performance of existing enzymes for biotechnological applications.

*Rhynchophorus palmarum* (*R. palmarum*) is an insect pest widely distributed in the tropical regions and in agrosystems exploiting oil palms [6]. Owing to their diverse complex carbohydrate-degrading enzymes [8–12], natural abundance, large biomass, and rearing feasibility, *R. palmarum* larvae represent prime candidates for bioprospecting.

Previous studies have reported the biochemical characterization of *β*-galactosidase from *R. palmarum* larvae [11]. Interesting results were also obtained using a synthetic substrate, *p*-nitrophenyl-*β*-D-galactopyranoside (*p*NPG). These studies also investigated the influence of pH and temperature on enzyme activity which are among the important factors in the control of bioprocesses in biotechnology [6,13–15]. However, no comprehensive physicochemical modeling based on empirical and mechanistic approaches has been reported. Kinetic modeling allows the determination of key parameters such as activation energy (E_a_), essential for assessing enzyme efficiency in industrial bioprocesses. Key kinetic parameters, including K_m_, V_max_, k_cat_ and catalytic efficiency (k_cat_/K_m_), provide essential information on substrate affinity and catalytic performance. Accurate estimation of these parameters through empirical and mechanistic modeling allows for a more reliable characterization of the enzyme and supports the rational optimization of enzyme-based biotechnological processes [15]. Therefore, investigating the kinetic and physicochemical properties of *β*-galactosidase from *R. palmarum* is essential for understanding its catalytic behavior and assessing its biotechnological potential. In this work, enzymatic hydrolysis of *o*-nitrophenyl-*β*-D-galactopyranoside (*o*NPG) in the presence of *β*-galactosidase from *R. palmarum* was performed. In this context, the present study aims to provide a comprehensive physicochemical and kinetic characterization of this enzyme using empirical, mechanistic models and nonlinear regression. The effects of temperature and pH on enzyme activity were examined and key kinetic parameters were determined. This integrated approach provides a more accurate description of enzyme behaviour and provides valuable insights for potential biotechnological applications.

## 2. Materials and methods

### 2.1. Chemicals

*o*NPG and all other analytical grade reagents were purchased from Merck KGaA® (Darmstadt, Germany). Chromatographic media, including DEAE-Sepharose CL-6B, Sephacryl S-100 HR and Phenyl Sepharose CL-6B, were obtained from Pharmacia-LKB Biotech (Uppsala, Sweden). All the chemicals used were of analytical grade and from Merck KGaA®.

### 2.2. Enzyme samples

Enzymatic crude extract was obtained following the method described by Yapi et al. [11]. *R. palmarum* larvae were rinsed with distilled water and wrung out on filter paper. The digestive tract was isolated in potassium chloride solution (0.9%; w/v) using forceps and emptied of its contents. The digestive juice thus obtained was diluted (1/1; v/v) in a potassium chloride solution (0.9%; w/v). Centrifugation was then carried out at 6000 rpm for 30 min at 277.15 K, using a UNICEN ALRESA brand refrigerated centrifuge. The supernatant obtained was diluted (1/1; v/v) in the sodium acetate buffer at 100 mM pH = 5.6 and centrifuged again at 9000 rpm under the same conditions. The final supernatant was used as the crude enzyme extract.

### 2.3. Enzymatic assay

*β*-galactosidase activity was measured using a UV-visible spectrophotometer (Pioway Medical Lab. Equipment Co., Ltd. 5100). The reaction mixture contained 75 µL of 5 mM *o*NPG, 150 µL of sodium acetate buffer (100 mM, pH = 5.6) and 50 μL of enzyme solution. The reaction was incubated at 310.15 K for 10 min and stopped by adding 3 mL of 1 M sodium carbonate. Absorbance was measured at 420 nm. A control without enzyme was included. One unit (U) of enzyme activity was defined as the amount of enzyme required to release 1 μmol of *o*-nitrophenol (*o*NP) per minute under the assay conditions. Specific activity was expressed as U mg ^−1^ of protein.

### 2.4. Protein assay and purification procedures

Protein concentration was measured using Lowry et al. [16] method, with bovine serum albumin as the standard. The purification was carried out in three chromatographic steps involving anion exchange chromatography, molecular exclusion gel fractionation and hydrophobic interaction chromatography (see Yapi et al. [11] for further details).

### 2.5. Influence of temperature and pH on enzyme activity

#### 2.5.1. Experimental.

The influence of pH on enzyme activity is evaluated through assays conducted at 310.15 K using sodium acetate buffer (100 mM, 3.6 ≤ pH ≤ 5.6) and sodium phosphate buffer (100 mM, 5.6 ≤ pH ≤ 8.0). The influence of temperature is examined by measuring enzyme activity at pH = 5.6 (100 mM sodium acetate buffer) over a temperature range of 303.15 K to 353.15 K.

#### 2.5.2. Models describing the effect of temperature on *R. palmarum β*-galactosidase activity.

When *β*-galactosidase activity is plotted against temperature, a bell-shaped curve is observed: activity increases with rising temperature, reaches a peak, and then declines [11]. Several empirical and mechanistic models developed for biological growth rates [17–19] can be applied to study the effect of temperature on enzyme activity. For more details, see the previous work [20].

Empirical models (Eqs. (1) and (2)) and mechanistic models (Eq. (3)), which explicitly determine T_opt_, were employed to analyze the experimental data.

- Cardinal temperature model with inflection (CTMI) by Rosso et al. [21,22]

$$\begin{array}{c} \text{A}(\text{T})={\text{A}}_{\text{opt}}^{\text{T}}\frac{\left(\text{T}-{\text{T}}_{\text{max}}\right){\left(\text{T}-{\text{T}}_{\text{min}}\right)}^{\text{2}}}{\left({\text{T}}_{\text{opt}}-{\text{T}}_{\text{min}}\right)\left\{\left({\text{T}}_{\text{opt}}-{\text{T}}_{\text{min}}\right)\left(\text{T}-{\text{T}}_{\text{opt}}\right)-\left({\text{T}}_{\text{opt}}-{\text{T}}_{\text{max}}\right)\left({\text{T}}_{\text{opt}}+{\text{T}}_{\text{min}}-\text{2T}\right)\right\}} \end{array}$$ (1)

- Where, ${\text{A}}_{\text{opt}}^{\text{T}}$ is the highest activity at optimum temperature T_opt_.
- Blanchard model (BM) [14–17]

$$\begin{array}{c} \text{A}(\text{T})={\text{A}}_{\text{opt}}^{\text{T}}{\left(\frac{{\text{T}}_{\text{max}}-\text{T}}{{\text{T}}_{\text{max}}-{\text{T}}_{\text{opt}}}\right)}^{\beta }\text{exp}\left(-\beta \frac{{\text{T}}_{\text{opt}}-\text{T}}{{\text{T}}_{\text{max}}-{\text{T}}_{\text{opt}}}\right) \end{array}$$ (2)

- With *β* the Blanchard parameter.
- Wojcik and Miłek model (WMM) [18]

$$\begin{array}{c} \text{A}(\text{T})={\text{A}}_{\text{opt}}^{\text{T}}\frac{\text{exp}\left(\frac{\left({\text{T}}_{\text{opt}}-\text{T}\right){\text{E}}_{\text{d}}\beta }{\text{RT}{\text{T}}_{\text{opt}}(\text{exp}\beta -\text{1})}\right)\left\{\text{1}-\text{exp}\left[-\beta \text{exp}\left(\frac{{\text{E}}_{\text{d}}\left(\text{T}-{\text{T}}_{\text{opt}}\right)}{\text{RT}{\text{T}}_{\text{opt}}}\right)\right]\right\}}{\text{1}-\text{exp}(-\beta )} \end{array}$$ (3)

- *R* is the gas constant, T is the absolute temperature in Kelvin, E_d_ is the activation energy for enzyme deactivation, *β* is the Wojcik and Miłek parameter.
- The activation energy E_a_ is given by (Eq. (4)):

$$\begin{array}{c} {\text{E}}_{\text{a}}={\text{E}}_{\text{d}}-\frac{{\text{E}}_{\text{d}}\beta }{\text{exp}\beta -\text{1}} \end{array}$$ (4)

- Alexandrov and Yamagata model (AYM) [19]

$$\begin{array}{c} \text{A}(\text{T})={\text{A}}_{\text{opt}}^{\text{T}}\frac{{\text{H}}_{\text{d}}\text{exp}(\frac{{\text{H}}_{\text{a}}\left(\text{T}-{\text{T}}_{\text{opt}}\right)}{\text{TR}{\text{T}}_{\text{opt}}})}{{\text{H}}_{\text{d}}-{\text{H}}_{\text{a}}(\text{1}-\text{exp}(\frac{{\text{H}}_{\text{d}}\left(\text{T}-{\text{T}}_{\text{opt}}\right)}{\text{TR}{\text{T}}_{\text{opt}}}))} \end{array}$$ (5)

- Where T is absolute temperature, H_a_ and H_d_ are interpreted as the levels of activation energy below and above enzyme’s optimal temperature (T_opt_), respectively.

#### 2.5.3. Models describing the effect of pH on *β*-galactosidase activity.

As observed for temperature, the effect of pH on *β*-galactosidase activity follows a characteristic bell-shaped curve [11]. This behavior, where activity increases from near zero in highly acidic conditions to a maximum value before dropping back to zero in strongly alkaline conditions, is common to most enzymes [23]. Considering that only the native enzyme remains catalytically active, it has been demonstrated that enzyme activity under substrate-saturation conditions can be influenced by pH according to Eqs. (7) and (8) [24,25].

All models (mechanistic and empirical) employed in this study to describe the relationship between enzyme activity and pH are presented below.

- Cardinal pH model with inflection (CPMI) by Rosso et al. [21,22,26]
- To improve convergence of non-linear regression, Dantigny et al. [26] modified the original equation by swapping pH_min_ and pH_max_. This revised expression is adopted in the present study.

$$\begin{array}{c} \text{A}(\text{pH})={\text{A}}_{\text{opt}}^{\text{pH}}\frac{\left(\text{pH}-{\text{pH}}_{\text{min}}\right){\left(\text{pH}-{\text{pH}}_{\text{max}}\right)}^{\text{2}}}{\left({\text{pH}}_{\text{opt}}-{\text{pH}}_{\text{max}}\right)\left\{\left({\text{pH}}_{\text{opt}}-{\text{pH}}_{\text{max}}\right)\left(\text{pH}-{\text{pH}}_{\text{opt}}\right)-\left({\text{pH}}_{\text{opt}}-{\text{pH}}_{\text{min}}\right)\left({\text{pH}}_{\text{opt}}+{\text{pH}}_{\text{max}}-\text{2pH}\right)\right\}} \end{array}$$ (6)

- Mechanistic models (MeM) [24,25]:

$$\begin{array}{c} \text{A}(\text{pH})=\frac{{\text{V}}_{\text{max}}^{\text{opt}}}{\text{1}+{\text{10}}^{{\text{pK}}_{\text{ES1}}-\text{pH}}+{\text{10}}^{\text{pH}-{\text{pK}}_{\text{ES2}}}}\;(\text{MeM1}) \end{array}$$ (7)

$$\begin{array}{c} \text{A}(\text{pH})=\frac{{\text{V}}_{\text{max}}^{\text{opt}}}{\text{1}+{\text{10}}^{{\text{pK}}_{\text{ES1}}-\text{2pH}}+{\text{10}}^{\text{pH}-{\text{pK}}_{\text{ES2}}}}\;(\text{MeM2}) \end{array}$$ (8)

- ${\text{V}}_{\text{max}}^{\text{opt}}$ represents the maximum velocity (activity) at optimum pH.

### 2.6. Kinetic parameters determination

Kinetic parameters of *β*-galactosidase from *R. palmarum* larvae were determined by measuring the initial velocity (V₀) of *o*NPG hydrolysis at various substrate concentrations ranging from 0 to 2.3 mM in the standard reaction mixture (at 310.15 K in 100 mM sodium acetate buffer, pH = 5.6). The apparent Michaelis constant (K_m_) and maximum velocity (V_max_) were estimated using the hyperbolic Michaelis-Menten equation (Eq. (9)), as well as three linearized models: Lineweaver-Burk plot (Eq. (10)), Eadie-Hofstee plot **(**Eq. (11)), and Hanes-Woolf plot (Eq. (12)).


$$\begin{array}{c} {\text{V}}_{\text{0}}=\frac{{\text{V}}_{\text{max}}[\text{S}]}{{\text{K}}_{\text{m}}+[\text{S}]} \end{array}$$ (9)

$$\begin{array}{c} \frac{\text{1}}{{\text{V}}_{\text{0}}}=\frac{{\text{K}}_{\text{m}}}{{\text{V}}_{\text{max}}}\times \frac{\text{1}}{\left[\text{S}\right]}+\frac{\text{1}}{{\text{V}}_{\text{max}}} \end{array}$$ (10)

$$\begin{array}{c} {\text{V}}_{\text{0}}=-{\text{K}}_{\text{m}}\frac{{\text{V}}_{\text{0}}}{\left[\text{S}\right]}+{\text{V}}_{\text{max}} \end{array}$$ (11)

$$\begin{array}{c} \frac{\left[\text{S}\right]}{{\text{V}}_{\text{0}}}=\frac{\text{1}}{{\text{V}}_{\text{max}}}\times [\text{S}]+\frac{{\text{K}}_{\text{m}}}{{\text{V}}_{\text{max}}} \end{array}$$ (12)

Where V_0_ is the initial velocity of reaction (U mg^-1^) and [S] is the concentration of substrate (mM).

### 2.7. Statistical analysis and non-linear regression

To determine statistical significance, a one-way analysis of variance (ANOVA) was performed at a significance level of 0.05. If the ANOVA indicates a statistically significant result, a Duncan post-hoc test is conducted. All assays were performed in at least three independent replicates, and the results are reported as mean ± standard deviation (SD). Statistical analyses were conducted using IBM SPSS software, version 22. Non-linear regressions were performed using SigmaPlot version 15.0 with 200 iterations, a step size of 1 and a tolerance of 10^-12^.

## 3. Results and discussion

### 3.1. Purification of *β*-galactosidase

The purification process of *β*-galactosidase from *R. palmarum* is achieved to a level of 86.88-fold purification, yielding an overall enzyme yield of 2.22% and a specific activity of 28.67 U mg^-1^ (see Table 1). These values fall within the range reported for insect *β*-galactosidase (23.84 to 39.73) U mg^-1^ [11,27], indicating that the enzyme preparation is comparable to those described in literature. However, as reported by Fuerst et al. [28], a purification factor of 5200 and a specific activity of 780 U mg^-1^ was achieved for *β*-galactosidase from *Drosophila melanogaster*. The results obtained demonstrate that *R. palmarum* larvae represent a significant biological source of glycosidases which possess noteworthy catalytic properties.

**Table 1:** Summary of purification of *β*-galactosidase from *R. palmarum.*

Purification steps	Total activity (U)a	Total protein (mg)	Specific activity (Umg-1)	Purification factor	Yield (%)
**Crude extract**	19.35	59.30	0.33	1	100
**DEAE Sepharose CL-6B**	2.76	4.19	0.66	2	14.26
**Ammonium sulfate precipitation (80% saturation)**	1.13	0.78	1.45	4.40	5.84
**Sephacryl S-100 HR**	1.09	0.38	2.87	8.70	5.63
**Phenyl-Sepharose CL-4B**	0.43	0.01	28.67	86.88	2.22

^a^U: One unit equals 1 µmol of *o*NP release per min (pH = 5.6; T = 310.15 K).

### 3.2. Influence of temperature on *β*-galactosidase activity

**Fig 1:**
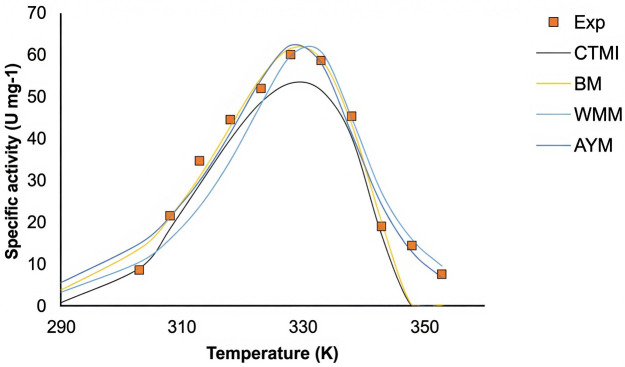
Activity of *β*-galactosidase from *R. palmarum* versus temperature (Models compared to experimental data).

The temperature dependence of *β*-galactosidase activity exhibited a typical bell-shaped profile (Fig 1), reflecting the balance between increasing catalytic rates and thermal denaturation at elevated temperatures [29,30]. The optimal temperature (T_opt_ = 330 K) (Table 2) was consistently estimated across all models, thereby demonstrating the robustness of the fitting approaches. Despite the superior statistical compatibility of empirical models, mechanistic models were favored on account of their enhanced biochemical pertinence [20], thus ensuring a higher degree of realism. Empirical models provide a superior statistical fit (higher R^2^ and lower AICc), with their parameters primarily describing the mathematical form of the activity profile. Consequently, these parameters should be interpreted as descriptive quantities. For instance, T_min_, T_max_, pH_min_, and pH_max_ delineate the activity limits, while the Blanchard and Wojcik-Miłek coefficients principally regulate the asymmetry and slope of the curve, without direct physicochemical significance. In contrast, mechanistic models are based on the kinetic and thermodynamic principles of enzymes. Consequently, the estimated parameters are associated with explicit physicochemical significance: E_a_ reflects the energy barrier associated with catalysis, E_d_ is representative of thermal inactivation, and pK_ES1_ and pK_ES2_ correspond to the apparent ionization constants of the catalytic residues in the enzyme-substrate complex. While mechanistic models may occasionally exhibit a slightly lower statistical fit than purely empirical models, their parameters are interpretable within biological context. Consequently, mechanistic models are more appropriate for the comprehension of enzyme function and for purpose of comparison with values documented in the enzymatic literature.

**Table 2:** Estimated parameters from different models used to fit enzyme activity of *β*-galactosidase from *R. palmarum* versus temperature (at pH = 5.6).

Empirical models		Mechanistic models		
Parameters	^a^CTMI	^b^BM	^c^WMM	^d^AYM
${𝐀}_{𝐨𝐩𝐭}^{𝐓}$ **(U mg**^**-1**^)	53.6 ± 0.9	62.0 ± 2.0	62.3 ± 2.9	62.7 ± 2.8
**T**_**opt**_ **(K)**	329.4 ± 0.4	329.5 ± 0.8	330.9 ± 1.5	329.1 ± 0.9
**T**_**min**_ **(K)**	292.8 ± 1.4	---	---	---
**T**_**max**_ **(K)**	345.4 ± 0.3	353.1 ± 5.9	---	---
**β(Blanchard)**	---	4.1 ± 2.2	---	---
**E**_**a**_ **(kJ mol**^**-1**^)	---	---	54 ± 8	56.3 ± 9.0
**E**_**d**_ **(kJ mol**^**-1**^)	---		171.2 ± 16.9	197.2 ± 19.3
**β(WMM)**	---	---	0.7 ± 0.2	---
**Statistic** ^**c**^				
**R** ^**2**^	0.99	0.98	0.97	0.97
$R_{adj}^{2}$ ^**e**^	0.99	0.97	0.96	0.95
**AICc**	33.15	45.69	48.08	48.56
**PRESS**	36.32	293.64	291.09	297.01
**F-test**	352.54	86.21	76.00	72.67

^a^CTMI: Cardinal Temperature Model with Inflection; ^b^BM: Blanchard Model; ^c^WMM: Wojcik and Miłek Model; ^d^AYM: Alexandrov and Yamagata Model; ^e^Adjusted R-squared; AICc: Corrected Akaike Information Criterion; PRESS: Predicted Residual Sum of Squares; F-test: Fisher’s coefficient.

The relatively high optimal temperature suggests that the enzyme possesses notable thermotolerance, which is advantageous for industrial applications requiring elevated temperatures in order to enhance reaction rates and limit microbial contamination. This optimum is notably high for an insect-derived *β*-galactosidase. While this optimum exceeds the typical body temperature of the larvae, it demonstrates notable thermotolerance, which is likely indicative of an evolutionary adaptation to the exothermic conditions encountered when degrading plant tissues. Furthermore, studies by Sharifi et al. [7] on *β*-galactosidase extracted from the digestive tract of *Xanthogaleruca luteola* demonstrated an optimum temperature of 60 °C (333 K). In contrast, the optimal temperature for *β*-galactosidase from *R. ferrugineus* is 40 °C (313 K) [6]. With regard to catalytic performance, the *β*-galactosidase from *R. palmarum* larvae exhibited an optimal temperature of 330.0 K (57 °C), thus demonstrating remarkably high thermal tolerance in comparison to several recently characterized microbial homologs. For instance, the modified *β*-galactosidase from *Bifidobacterium longum* BIM B-813 D reported by Morozova et al. [31] had an optimal temperature range extending up to 50 °C. The comparatively higher optimum temperature observed for the *R. palmarum* enzyme highlights its robust catalytic capacity under high-temperature conditions, making it a promising candidate for biotechnological processes where temperature fluctuations occur.

The activation energy (E_a_ = 56.3 kJ. mol^-1^), determined through mechanistic modeling, has been found to be higher than the previously reported value (35.43 kJ. mol^-1^) [11]. This discrepancy is hypothesized to be attributable to the implementation of nonlinear regression analysis in the current study. This methodological approach provides more reliable parameter estimates than classical linearization techniques [20]. Indeed, the strength of the nonlinear regression approach used in this study, compared to traditional linearization of the Arrhenius plot, lies in its statistical robustness. Linearization methods frequently result in the distortion of the experimental error structure, thereby conferring an exaggerated significance upon specific data points [20].

### 3.3. Influence of pH on *β*-galactosidase activity

**Fig 2:**
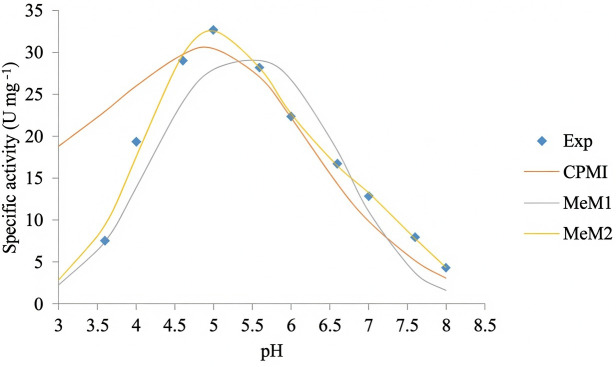
Activity of *β*-galactosidase from *R. palmarum* versus pH (Models compared to experimental data).

The pH-activity profile exhibited a bell-shaped curve (Fig 2), which is characteristic of enzyme systems involving ionizable catalytic residues [11,20]. The optimal pH (pH_opt_ = 5.0) (Table 3) is consistent with acidic digestive environments and aligns with previous findings for insect *β*-galactosidases [11]. The reported pH_opt_ values for *β*-galactosidase from *R. ferrugineus* (pH = 4.0) [6] and *Xanthogaleruca luteola* (pH = 3.0) [7] were lower than those determined in this study. This indicates that the *R. palmarum* enzyme functions optimally under less acidic conditions. In general, the pH_opt_ of *β*-galactosidases from different sources varies from 1.5 to 7.0 [32–36]. Furthermore, *β*-galactosidases from *R. palmarum* and *Bifidobacterium longum* BIM B-813 D share a similar acidic optimal pH profile (pH = 5.0), reflecting a conserved adaptation of glycosyl hydrolases functioning in specific acidic microenvironments, whether in the specialized digestive tract of insect larvae or in specific bacterial metabolic niches [31].

**Table 3:** Estimated parameters from different models used to fit enzyme activity of *β*-galactosidase from *R. palmarum* versus pH (T = 310.15 K).

	Empirical models	Mechanistic models	
Parameters	CPMI^(a)^	MeM1^(b)^	MeM2^(c)^
${𝐀}_{𝐨𝐩𝐭}^{𝐩𝐇}$ **(U mg**^**-1**^)	30.5 ± 0.8	---	---
**pH**_**min**_	3.46 ± 0.04	---	---
**pH**_**opt**_^**(d)**^	4.93 ± 0.07	5.30 ± 0.20^(d)^	5.10 ± 0.10
**pH**_**max**_	9.1 ± 0.2	---	---
${𝐕}_{𝐨𝐩𝐭}^{𝐩𝐇}$ **(U mg**^**-1**^)	---	32 ± 3	36 ± 3
**pK**_**ES1**_	---	3.9 ± 0.2	4.0 ± 0.1
**pK**_**ES2**_	---	6.7 ± 0.2	6.2 ± 0.2
**Statistic**			
**R** ^**2**^	0.98	0.90	0.96
$R_{adj}^{2}$ ^**(e)**^	0.98	0.88	0.94
**AICc**	26.47	36.96	37.08
**PRESS**	101.79	159.92	97.45
**F-test**	150.96	34.59	50.93

(a) CPMI: cardinal pH model with inflection; (b) MeM1: Mechanistic model 1; (c) MeM2: Mechanistic model 2. (d) pH_opt_ = (pK_ES1_ + pK_ES2_)/2. (e): Adjusted R-squared; AICc: corrected Akaike information criterion; PRESS: Predicted residual sum of squares statistic; F-test: Fisher’s coefficient.

The estimation of the apparent ionization constants of the enzyme-substrate complex was conducted through the utilization of mechanistic modeling (pK_ES1_ = 4.0 and pK_ES2_ = 6.2). In accordance with the well-established catalytic mechanism of *β*-galactosidases and other glycoside hydrolases, catalysis necessitates the presence of two catalytic carboxylate residues: a nucleophile that must remain deprotonated and a general acid/base catalyst (proton donor) that must remain protonated during the cleavage of the glycosidic bond. Consequently, the lower apparent pK_ES_ value corresponds to the catalytic nucleophile, while the higher value corresponds to the proton donor. Despite the indirect nature of these attributions, which is predicated upon a mechanistic interpretation as opposed to direct structural evidence, a full consistency with the reported catalytic mechanism for glycosidase retention is demonstrated [37,38].

From a biotechnological perspective, the optimal pH level for the activity of *β*-galactosidase from *R. palmarum* (pH_opt_ = 5.0) is advantageous for industrial applications requiring enzyme activity under acidic conditions. Such applications include the hydrolysis of lactose in fermented dairy products (e.g., yogurt, pH = 4.0–4.5) or fruit-based beverages [39–41]. The specific activities of the enzyme (based on the best-fit model) at pH = 4 and 4.5 are 19.36 and 29.7 U mg^-1^, respectively. It is evident that acid-active *β*-galactosidases hold considerable industrial significance, primarily due to their ability to obviate the necessity for pH adjustment, thereby mitigating the risk of microbial contamination during processing [42].

### 3.4. Kinetic parameters

**Fig 3:**
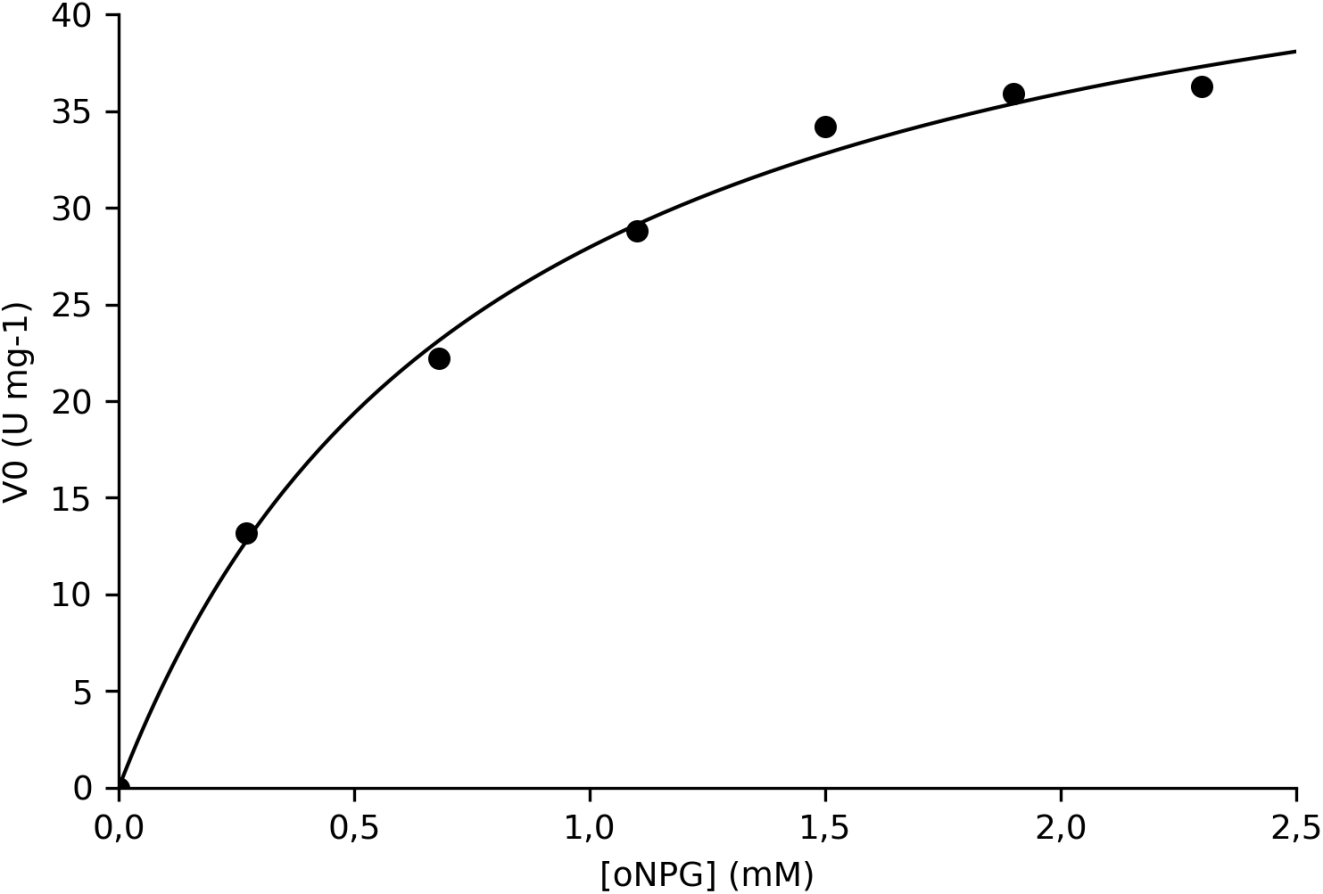
Michaelis-Menten plot (V_0_ versus [*o*NPG]) superimposed with experimental data.

**Fig 4:**
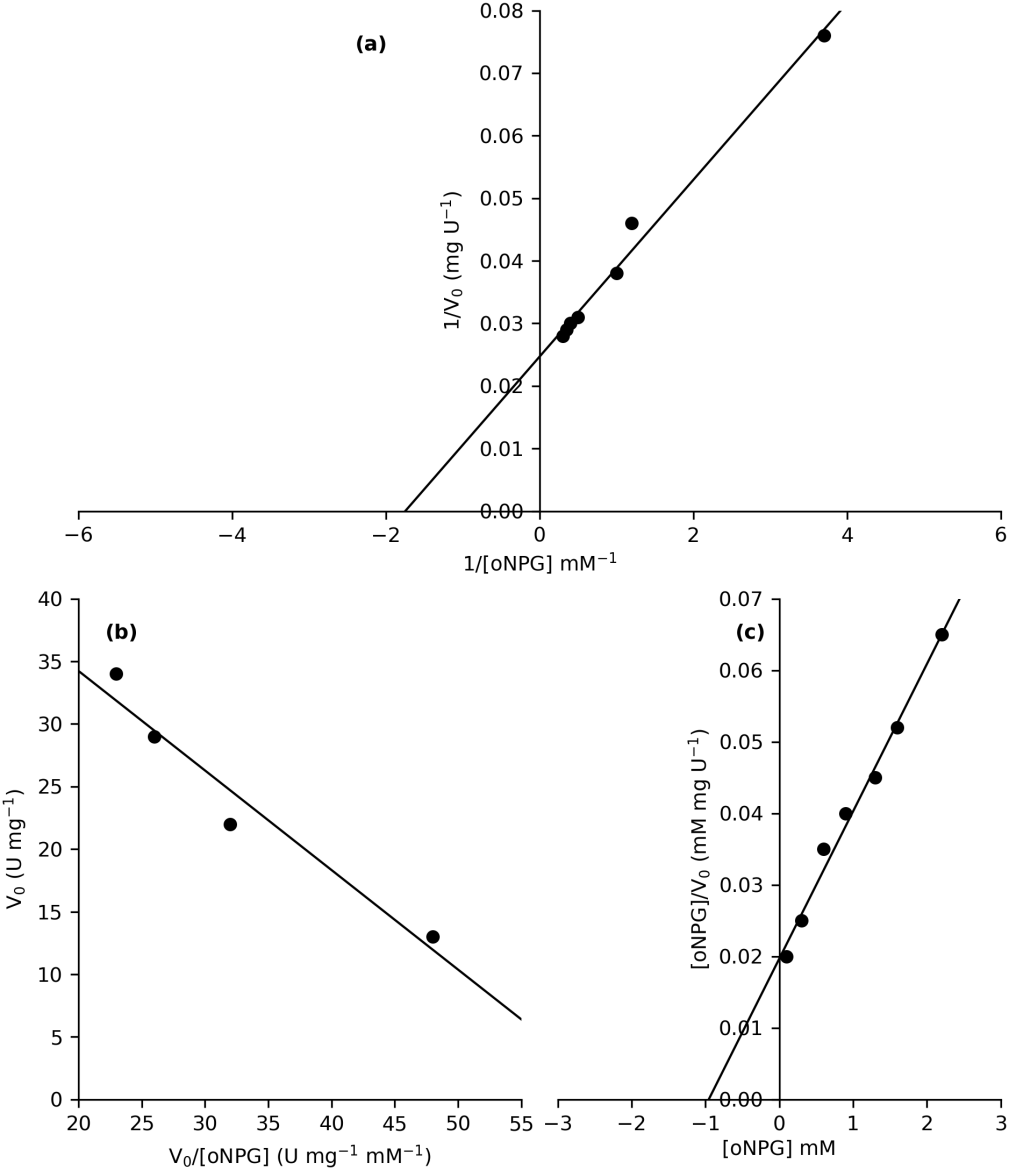
Linearized plots of Michaelis-Menten equation superimposed with experimental data: (a) Lineweaver-Burk plot (1/V_0_ versus 1/[*o*NPG]); (b) Eadie-Hofstee plot (V_0_ versus V_0_/[*o*NPG]); (c) Hanes-Woolf plot ([*o*NPG]/V_0_ versus [*o*NPG]).

The kinetic parameters determined in this study indicate a high catalytic efficiency of *β*-galactosidase from *R. palmarum* (Table 4). The corresponding graphical representations can be found in Figs 3 and 4. No evidence of enzymatic inhibition was observed. The K_m_ value (0.77 mM) reflects a strong affinity for the synthetic substrate *o*NPG, while the V_max_ (49 U mg ^−1^) indicates a high catalytic capacity. The values obtained were consistent across both nonlinear and linearized models, thereby confirming the reliability of the estimates. However, it should be noted that K_m_ value reflects the affinity for a synthetic substrate and may differ from that observed with natural substrates in vivo. The K_m_ value obtained in the present study is significantly lower than that proposed by Yapi et al. [11] (0.90 mM), which indicates a relatively high enzyme-substrate affinity in the present study. This discrepancy can be primarily attributed to the use of divergent test conditions, particularly with regard to substrate concentrations. Furthermore, when using insect larvae as an enzyme source, the K_m_ may also depend on the age (maturity) of the larvae [43–45] and on the organ or tissue (midgut, salivary gland, liver, etc.) [46,47].

**Table 4:** Kinetic parameters obtained in this work at 310.15 K and pH = 5.6.

	K_m_ (mM)	V_max_ (U mg ^−1^)	R^2^
**Michaelis-Menten**	0.80 ± 0.10	50 ± 2	0.99
**Lineweaver-Burk**	0.72 ± 0.06	48 ± 2	0.99
**Eadie-Hofstee**	0.75 ± 0.06	49 ± 2	0.97
**Hanes-Woolf**	0.80 ± 0.10	49 ± 2	0.99
**Mean value**	0.77 ± 0.08	49 ± 2	---

A review of the literature reveals an absence of studies on kinetic parameters (K_m_ and V_max_) of insect-derived *β*-galactosidases. When considering non-insect sources, reported K_m_ values range from 1.77 to 6.5 mM, while V_max_ values vary between 0.073 U mg^-1^ and 49.3 U mg^-1^ [48–53]. The parameters K_m_ and V_max_ are conventionally employed to characterize the enzyme-substrate affinity and the maximum catalytic capacity of an enzyme, in accordance with the Michaelis-Menten model [54–56]. The catalytic constant (k_cat_ = 4.9 × 10^3^ s^-1^) and catalytic efficiency (k_cat_/K_m_ = 6.36 × 10^6^ M^-1^ s^-1^) of this enzyme are indicative of its place among highly efficient biocatalysts, approaching diffusion-controlled limits [57]. In comparison with *β*-galactosidases from non-insect sources, the enzyme demonstrates higher affinity for the substrate, emphasizing its potential for industrial applications [57].

A limitation of this study is that the kinetic and mechanistic analyses were performed exclusively using *o*NPG, a synthetic chromogenic substrate commonly used for *β*-galactosidase assays. Although *o*NPG has been shown to provide a reliable and sensitive means of characterizing enzyme activity and estimating kinetic parameters, it has been demonstrated that its catalytic behavior may not fully reflect that observed with the natural substrate, lactose, or with other galactosides.

## Conclusion

The present study provides a physicochemical and kinetic characterization of *β*-galactosidase from *Rhynchophorus palmarum* larvae using advanced statistical modeling. The integration of empirical and mechanistic approaches in conjunction with nonlinear regression facilitated precise estimation of pivotal catalytic parameters, thereby unveiling an optimal temperature of 330.0 K and an optimal pH of 5.0. Mechanistic modeling further allowed determination of the apparent ionization constants of the enzyme–substrate complex (pK_ES1_ = 4.0 and pK_ES2_ = 6.2), thereby offering indirect insights into the protonation states of catalytic residues at the active site. A key limitation of this work is the exclusive use of the synthetic substrate *o*-nitrophenyl-*β*-D-galactopyranoside. It is recommended that future studies incorporate natural substrates such as lactose to validate these findings under more realistic conditions. In view of the high catalytic efficiency (k_cat_ = 4.9 × 10^3^ s^-1^), thermotolerance, and activity under mildly acidic conditions, immobilization of the enzyme on solid supports is a relevant perspective for the assessment of its stability and reusability in industrial applications. The results of this study demonstrate that insect digestive systems are a promising and underutilized source of robust enzymes for biotechnological applications.

## Supporting information

S1 Table Tables of raw experimental data for physicochemical and kinetic characterization of *β*-galactosidase from *R. palmarum.* (XLSX)

S2 Data Model equations and optimized parameters used for curve fitting of *β*-galactosidase activity from *R. palmarum* larvae as a function of temperature and pH. (XLSX)

S3 Data Data for linearized Michaelis-Menten plots (Lineweaver-Burk, Eadie-Hofstee, and Hanes-Woolf) of *β*-galactosidase from *R. palmarum.* (XLSX)

S4 File Purification procedure and SDS-PAGE analysis of purified *β*-galactosidase from *R. palmarum.* (DOCX)

S5 File Raw SDS-PAGE image of purified *β*-galactosidase from *R. palmarum.* (PNG)

S6. File SDS-PAGE profile of purified *β-*galactosidase from *R. palmarum.* **Note:** The SDS-PAGE experiment was performed in 2021 as part of the enzyme purification work. Given the time elapsed, the original uncropped acquisition file could not be retrieved despite our efforts.
(DOCX)
