# Supplementary material for: Kinetic and physicochemical modeling of β-galactosidase from Rhynchophorus palmarum larvae
Source: PLoS One. 2026 Jul 22;21(7):e0354469. doi: 10.1371/journal.pone.0354469 (PMC13390822; doi:10.1371/journal.pone.0354469)
Supplement: S4 File — (DOCX) [file pone.0354469.s004.docx]

**S4-Purification procedure and SDS-PAGE analysis of purified *β*-galactosidase from R. palmarum**

1. **Purification procedures**

**Purification** was carried out through **three chromatographic steps,** including **anion exchange chromatography, size-exclusion chromatography, and hydrophobic interaction chromatography** (see **Yapi et al. [1]** for further details). The **purity and molecular weight** of the purified ***β*-galactosidase** were evaluated by **sodium dodecyl sulfate-polyacrylamide gel electrophoresis (SDS-PAGE) [2],** employing a **10 % (w/v) separating gel** and a **4 % (w/v) stacking gel** (Hoefer mini-gel system; [www.hoeferinc.com](http://www.hoeferinc.com)). Protein bands are visualized using **silver staining**, following the protocol established by **Blum et al. [3]**. Before electrophoresis, the sample is **denatured at 373.15 K for 5 minutes**. The **molecular weight** of the purified enzyme was determined using **pre-stained molecular weight markers** (Bio-Rad).

1. **SDS-PAGE analysis**

The SDS-PAGE analysis of the purified *β*-galactosidase reveals a single band at approximately 60 ± 2 kDa (Fig.), which is consistent with the findings of Yapi et al. [1]. This result confirms that *R. palmarum* *β*-galactosidase has been purified to electrophoretic homogeneity and also suggests that the enzyme exists in a monomeric form, as previously reported [1]. To date, data on the molecular weights of insect *β*-galactosidases remain scarce in the scientific literature.

**References**

**1.** Yapi DYA, Niamké SL, Kouamé LP: Biochemical characterization of a strictly specific beta-galactosidase from the digestive juice of the palm weevil *Rhynchophorus palmarum* larvae, Entomological Science. 2007; 10: 343–352. https://doi.org/10.1111/j.1479-8298.2007.00232.x.

**2.** Wenk FAM**.** A manual for biochemistry protocols, manuals in biomedical research, in: Jan-Thorsten Schantz Series Editor. 2007; 3.

**3.** H. Blum, H. Beier, B. Gross, Improved silver staining of plant proteins, RNA and DNA in polyacrylamide gels, Electrophoresis 8 (1987) 93-99.
