# Supplementary figures and images for: Kinetic and physicochemical modeling of β-galactosidase from Rhynchophorus palmarum larvae
Source: PLoS One. 2026 Jul 22;21(7):e0354469. doi: 10.1371/journal.pone.0354469 (PMC13390822; doi:10.1371/journal.pone.0354469)

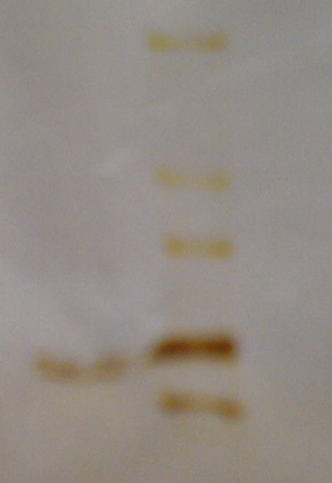

Supplement: S5 File — (PNG) [file pone.0354469.s005.png]
