# Supplementary material for: Kinetic and physicochemical modeling of β-galactosidase from Rhynchophorus palmarum larvae
Source: PLoS One. 2026 Jul 22;21(7):e0354469. doi: 10.1371/journal.pone.0354469 (PMC13390822; doi:10.1371/journal.pone.0354469)
Supplement: S6. File — Note: The SDS-PAGE experiment was performed in 2021 as part of the enzyme purification work. Given the time elapsed, the original uncropped acquisition file could not be retrieved despite our efforts. (DOCX) [file pone.0354469.s006.docx]

**S5- SDS-PAGE profile of purified *β-*galactosidase from *R. palmarum***

1 2


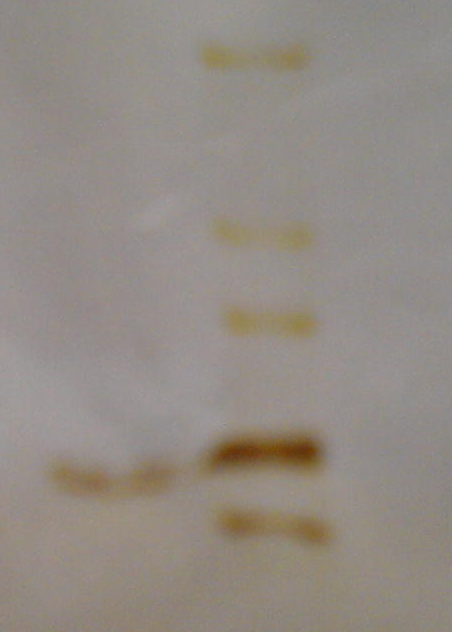


*β*-gal (60±2 kDa)

200 kDa

116,2 kDa

97,4 kDa

66,2 kDa

45 kDa

**Fig.** SDS-PAGE profile of purified *R. palmarum* *β-*galactosidase. Lane 1**:** Purified *R. palmarum* *β*-galactosidase (60±2 kDa). Lane 2**:** Molecular weight markers (Myosin, 200 kDa; β-Galactosidase, 116.25 kDa; Phosphorylase b, 97.4 kDa; Bovine Serum Albumin, 66.2 kDa; Ovalbumin, 45 kDa).
