# Supplementary material for: Kinetic and physicochemical modeling of β-galactosidase from Rhynchophorus palmarum larvae
Source: PLoS One. 2026 Jul 22;21(7):e0354469. doi: 10.1371/journal.pone.0354469 (PMC13390822; doi:10.1371/journal.pone.0354469)
Supplement: Attachment — Submitted filename: Response to Reviewers.docx [file pone.0354469.s008.docx]

**Response to Reviewers**

Manuscript ID: PONE-D-26-15402

Kinetic and physicochemical modeling of *β*-galactosidase from *Rhynchophorus palmarum* larvae

Dear Editor and Reviewers,

We would like to sincerely thank the Academic Editor and the reviewers for their careful evaluation of our manuscript and for their constructive comments. We greatly appreciate the time and effort devoted to improving the quality of our work.

We have carefully considered all comments and suggestions and have revised the manuscript accordingly. All modifications are highlighted (in red) in the revised manuscript. Detailed responses to each comment are provided in attached file (Response to reviewers).

We believe that the revised version has been significantly improved and hope that it is now suitable for publication in PLOS ONE.

Best regards,

The corresponding author.

**Reviewer #1**

**1-**The introduction devotes too much attention to the agricultural damage caused by *Rhynchophorus palmarum* and insufficient emphasis on its potential as a source of digestive enzymes.

**Response:**
We thank the reviewer for this valuable suggestion. The introduction has been revised to reduce the emphasis on the agricultural impact of *Rhynchophorus palmarum* and its role as a vector of red ring disease. Additional information has been incorporated regarding the digestive physiology of the larvae, their abundance, their large larval biomass, and their potential as a promising biological source of glycosidases. This modification strengthens the rationale for selecting this organism in the context of enzyme bioprospecting.

**2-**The large number of mathematical models may overwhelm readers.

**Response:**
We appreciate this comment. To improve readability, we have added a brief biochemical interpretation of each model in plain language. In addition, we have simplified the presentation of the modelling framework and reduced unnecessary mathematical details in the main text while retaining essential equations and parameter definitions.

**3-**The statement describing the enzyme as having "competitive catalytic properties" appears overstated.

**Response:**
We agree with the reviewer. The sentence has been revised to adopt a more balanced interpretation of the results. The expression “competitive catalytic properties” has been replaced by “promising catalytic properties” to better reflect the experimental evidence.

**4-**Provide actual activity values at pH 4.0 and 4.5.

**Response:**
Thank you for this suggestion. The discussion section has been expanded to include the relative activity values observed at pH 4.0 and pH 4.5 (19.36 and 29.7 U mg ⁻¹, respectively).

**5-**Improve consistency of abbreviations and significant figures.

**Response:**
The manuscript has been thoroughly revised to ensure consistency in abbreviations, nomenclature, and significant figures throughout the text, tables, and figures.

**Reviewer #2**

**1-**The specific research gap is not clearly articulated.

**Response:**
We thank the reviewer for this important observation. The introduction has been revised to clearly identify the existing knowledge gap. Previous studies on β-galactosidase from *Rhynchophorus palmarum* mainly focused on purification and basic biochemical characterization. However, no comprehensive physicochemical modelling based on empirical and mechanistic approaches has been reported. The objective and originality of the present study have been clarified accordingly.

**2-**Differences with Yapi et al. (2007) require further explanation.

**Response:**
We agree with the reviewer. The Discussion has been expanded to provide a more detailed comparison with Yapi et al. (2007). We now discuss the influence of parameter estimation methods, particularly nonlinear regression versus graphical linearization, on the resulting kinetic and physicochemical parameters. For some kinetic parameters as K_m_, we can say that the difference could be explained primarily by the fact that we did not use exactly the same test conditions, particularly with regard to substrate concentrations. Furthermore, when using insect larvae as an enzyme source, the K_m_ may also depend on the age (maturity) of the larvae and on the organ or tissue (midgut, salivary gland, liver, etc.).

**3-**Explain why mechanistic models are considered more reliable.

**Response:**

We thank the reviewer for this insightful comment. We have revised the discussion section to clarify the interpretation of parameter estimates obtained from empirical and mechanistic models. Empirical models are primarily mathematical descriptions designed to reproduce the experimental bell-shaped profiles of enzyme activity as a function of temperature or pH. Their parameters (T_min_, T_max_, pH_min_, pH_max_,…) are useful for precisely identifying optimal conditions and defining the range of activity, but they generally have no direct physicochemical significance. In contrast, mechanistic models are derived from enzyme kinetics and thermodynamic principles. Their parameters have explicit biochemical interpretations. Regarding temperature dependence, the estimated activation energy (E_a_) reflects the energy barrier of the catalytic process, while the deactivation energy (E_d_) quantifies the thermal inactivation of the enzyme. Regarding pH dependence, the estimated values of pK_ES1_ and pK_ES2_ correspond to the apparent pK values of the ionizable catalytic groups within the enzyme-substrate complex, thereby providing information on the protonation states required for catalysis. Although empirical models sometimes yielded a better statistical fit, mechanistic models were deemed more reliable for interpreting the parameters, as they are constrained by the underlying physicochemical mechanisms that govern enzymatic catalysis. Consequently, their estimated parameters are biologically relevant and can be directly compared to values reported for other enzymes, whereas empirical parameters primarily describe the shape of the curve without providing mechanistic information.

**4-**Interpretation of pKa values requires stronger support.

**Response:**

We agree that the assignment of apparent pK_ES_ values to the catalytic nucleophile and the acid/base proton donor must be supported by references. We have therefore revised the discussion section to clarify that these attributions are based on the well-established catalytic mechanism of retention glycoside hydrolases, particularly β-galactosidases. In these enzymes, the catalytic process involves two essential carboxylate residues: one acting as the catalytic nucleophile, which must remain deprotonated, and the other as a general acid/base catalyst (proton donor), which must remain protonated during the cleavage of the glycosidic bond. Consequently, the lowest apparent pK_ES_ value is assigned to the nucleophile, while the highest value is assigned to the proton donor. We have included the relevant reference, notably Davies and Henrissat (1995).

**5-**Only oNPG was used as substrate.

**Response:**

We thank the reviewer for this valuable comment. We agree that although oNPG is the standard chromogenic substrate widely used for the characterization of β-galactosidase due to its high sensitivity and convenient spectrophotometric detection, it does not fully represent the enzyme’s catalytic behavior toward its natural substrate, lactose. In the revised manuscript, we have explicitly acknowledged this limitation in the discussion (conclusion section). We also emphasize that future work will focus on validating kinetic and mechanistic models using lactose and other natural galactosides, as well as on evaluating the enzyme under conditions relevant to the industrial hydrolysis of lactose. These additions clarify the scope of the present work while outlining important directions for future research.

**6-**The manuscript focuses on optimal temperature but provides limited information on thermostability.

**Response:**

We fully agree with the reviewer’s pertinent comment. In this study, we focused exclusively on the short-term effect of temperature on enzymatic activity (thermotolerance) in order to optimize operational parameters through empirical and mechanistic modeling. Since we did not conduct time-dependent thermal inactivation assays (pre-incubation experiments), we cannot address the concept of “thermostability” in the strict kinetic sense. To avoid any misunderstanding and ensure absolute scientific rigor, we have carefully revised the manuscript. All instances of the terms “thermostability” and “thermal stability” have been replaced with “thermotolerance” or “thermal tolerance.”

**7-**The discussion on enzyme stability and catalytic activity should be strengthen by expanded comparative analysis with the recent reported data. For example, <https://doi.org/10.1016/j.ijbiomac.2026.152774>

**Response:**

We sincerely thank the reviewer for bringing this very recent and relevant study to our attention (Morozova et al., 2026, Int. J. Biol. Macromol.). We have carefully read the suggested article and have expanded our comparative analysis accordingly.

In the revised version of the manuscript, we have expanded the comparative analysis of the catalytic behavior of our enzyme with that of the modified β-galactosidase from *Bifidobacterium longum* BIM B-813 D. We have demonstrated that, while the *B. longum* enzyme functions effectively up to 50 °C, the β-galactosidase from *R. palmarum* larvae exhibits an even higher optimal temperature (57 °C / 330.0 K), highlighting its apparent exceptional thermotolerance. We also noted the interesting similarity in their optimal pH profiles (pH 5.0). This comparison significantly strengthens the biotechnological positioning of our biocatalyst.

**8-**Conclusions should be better supported by the key data obtained. Clearly state limitations and future perspectives.

**Response:**

We have thoroughly rewritten the Conclusion section to better emphasize our quantitative findings while explicitly addressing the limitations and future directions of this research.

Specifically, we have:

(i) summarized the key modeling and operational data (T_opt_ at 57 °C, pH = 5.0, WMM/AYM validation);

(ii) explicitly stated the limitation regarding the non-using of natural substrate (lactose);

(iii) outlined clear future perspectives, including enzyme purification, immobilization strategies, and industrial application trials (lactose hydrolysis).

**9-**The text should be carefully checked for typos and grammatical errors.

**Response:**

We appreciate this recommendation. The entire manuscript has been thoroughly reviewed and edited to correct typos, improve sentence structure, and ensure standard English scientific style throughout.

**Reviewer #3**

The manuscript requires careful proofreading and language editing to improve grammatical accuracy, clarity, and academic fluency. In addition, there are several typographical errors, punctuation inconsistencies, and minor formatting issues throughout the text that should be corrected. The authors should also ensure consistency in terminology, abbreviations, and sentence structure across the manuscript.

**Response:**

We greatly appreciate the reviewer’s careful evaluation and constructive suggestions. Following the reviewer’s recommendation, the manuscript underwent a comprehensive linguistic revision to improve grammatical accuracy, clarity, readability, and the fluidity of the writing style. Particular attention was paid to correcting typos, punctuation inconsistencies, formatting issues, and sentence structure. In addition, we carefully reviewed the manuscript to ensure consistency in terminology, abbreviations, symbols, units, significant figures, and writing style throughout all sections, tables, and figures. We are confident that these revisions have significantly improved the overall quality and presentation of the manuscript.
