# Supplementary material for: Kinetic and physicochemical modeling of β-galactosidase from Rhynchophorus palmarum larvae
Source: PLoS One. 2026 Jul 22;21(7):e0354469. doi: 10.1371/journal.pone.0354469 (PMC13390822; doi:10.1371/journal.pone.0354469)
Supplement: Peer review history

# Peer review: Decision Letter 0

**DOI:** 10.1371/journal.pone.0354469.r001

**Authors:** Rafael Vazquez-Duhalt

23 Jun 2026

PONE-D-26-15402

Kinetic and physicochemical modeling of β-galactosidase from *Rhynchophorus palmarum*  larvae

PLOS One

Dear Dr. KAMBIRE,

Thank you for submitting your manuscript to PLOS ONE. After careful consideration, we feel that it has merit but does not fully meet PLOS ONE’s publication criteria as it currently stands. Therefore, we invite you to submit a revised version of the manuscript that addresses the points raised during the review process.

==============================

Please address all the reviewers´ suggestions and comments, then submit the revised version.

==============================

Please submit your revised manuscript by Aug 07 2026 11:59PM. If you will need more time than this to complete your revisions, please reply to this message or contact the journal office at plosone@plos.org. When you're ready to submit your revision, log on to https://www.editorialmanager.com/pone/ and select the 'Submissions Needing Revision' folder to locate your manuscript file.

Please include the following items when submitting your revised manuscript:

- A letter that responds to each point raised by the academic editor and reviewer(s). You should upload this letter as a separate file labeled 'Response to Reviewers'.
- A marked-up copy of your manuscript that highlights changes made to the original version. You should upload this as a separate file labeled 'Revised Manuscript with Track Changes'.
- An unmarked version of your revised paper without tracked changes. You should upload this as a separate file labeled 'Manuscript'.

If you would like to make changes to your financial disclosure, please include your updated statement in your cover letter. Guidelines for resubmitting your figure files are available below the reviewer comments at the end of this letter.

If applicable, we recommend that you deposit your laboratory protocols in protocols.io to enhance the reproducibility of your results. Protocols.io assigns your protocol its own identifier (DOI) so that it can be cited independently in the future. For instructions see: https://journals.plos.org/plosone/s/submission-guidelines#loc-laboratory-protocols. Additionally, PLOS ONE offers an option for publishing peer-reviewed Lab Protocol articles, which describe protocols hosted on protocols.io. Read more information on sharing protocols at https://plos.org/protocols?utm_medium=editorial-email&utm_source=authorletters&utm_campaign=protocols.

As the corresponding author, your ORCID iD is verified in the submission system and will appear in the published article. PLOS supports the use of ORCID, and we encourage all coauthors to register for an ORCID iD and use it as well. Please encourage your coauthors to verify their ORCID iD within the submission system before final acceptance, as unverified ORCID iDs will not appear in the published article. *Only*  the individual author can complete the verification step; PLOS staff *cannot*  verify ORCID iDs on behalf of authors.

We look forward to receiving your revised manuscript.

Kind regards,

Rafael Vazquez-Duhalt, Ph.D.

Academic Editor

PLOS One

Journal Requirements:

When submitting your revision, we need you to address these additional requirements.

1. Please ensure that your manuscript meets PLOS ONE's style requirements, including those for file naming. The PLOS ONE style templates can be found at

https://journals.plos.org/plosone/s/file?id=wjVg/PLOSOne_formatting_sample_main_body.pdf and

https://journals.plos.org/plosone/s/file?id=ba62/PLOSOne_formatting_sample_title_authors_affiliations.pdf.

2. Please provide your Data availability statement in English language.

3. If the reviewer comments include a recommendation to cite specific previously published works, please review and evaluate these publications to determine whether they are relevant and should be cited. There is no requirement to cite these works unless the editor has indicated otherwise.

4. Please review your reference list to ensure that it is complete and correct. If you have cited papers that have been retracted, please include the rationale for doing so in the manuscript text, or remove these references and replace them with relevant current references. Any changes to the reference list should be mentioned in the rebuttal letter that accompanies your revised manuscript. If you need to cite a retracted article, indicate the article’s retracted status in the References list and also include a citation and full reference for the retraction notice.

Additional Editor Comments (if provided):

The three reviewers agree that your work could be published in PLOS ONE after minor revisions.

Please address all reviewers' comments and suggestions and submit the revised version.

[Note: HTML markup is below. Please do not edit.]

Reviewers' comments:

Reviewer's Responses to Questions

**Comments to the Author**

1. Is the manuscript technically sound, and do the data support the conclusions?

The manuscript must describe a technically sound piece of scientific research with data that supports the conclusions. Experiments must have been conducted rigorously, with appropriate controls, replication, and sample sizes. The conclusions must be drawn appropriately based on the data presented.

Reviewer #1: Yes

Reviewer #2: Yes

Reviewer #3: Yes

**********

2. Has the statistical analysis been performed appropriately and rigorously?

Reviewer #1: Yes

Reviewer #2: No

Reviewer #3: Yes

**********

3. Have the authors made all data underlying the findings in their manuscript fully available?

The PLOS Data policy requires authors to make all data underlying the findings described in their manuscript fully available without restriction, with rare exception (please refer to the Data Availability Statement in the manuscript PDF file). The data should be provided as part of the manuscript or its supporting information, or deposited to a public repository. For example, in addition to summary statistics, the data points behind means, medians and variance measures should be available. If there are restrictions on publicly sharing data—e.g. participant privacy or use of data from a third party—those must be specified.

Reviewer #1: Yes

Reviewer #2: Yes

Reviewer #3: Yes

**********

4. Is the manuscript presented in an intelligible fashion and written in standard English?

PLOS ONE does not copyedit accepted manuscripts, so the language in submitted articles must be clear, correct, and unambiguous. Any typographical or grammatical errors should be corrected at revision, so please note any specific errors here.

Reviewer #1: Yes

Reviewer #2: No

Reviewer #3: No

**********

5. Review Comments to the Author

Please use the space provided to explain your answers to the questions above. You may also include additional comments for the author, including concerns about dual publication, research ethics, or publication ethics. (Please upload your review as an attachment if it exceeds 20,000 characters)

Reviewer #1: In this work, Kambiré and colleagues provide a robust framework for characterizing the β‑galactosidase from Rhynchophorus palmarum by combining empirical and mechanistic models, together with nonlinear regression. The results are both novel and practically relevant. After minor revisions, the article is recommended for publication.

1. In the Introduction, I notice a few points that would benefit from revision. First, the phrase listing “microorganisms, plants, insects and animals” implies that insects are placed outside the animal kingdom, which is taxonomically imprecise; I would suggest rephrasing it as “microorganisms, plants, and animals (including insects)” or simply “microorganisms, plants and animals” and then specifically mentioning insects later, to avoid this logical overlap. More importantly, a considerable portion of the introduction is devoted to describing the agricultural damage caused by Rhynchophorus palmarum and its role as a vector of red ring disease. While this contextual background is useful, it overshadows the justification for choosing this insect as an enzyme source. To strengthen the rationale, I would encourage the authors to expand on the digestive enzyme potential of this species, with particular emphasis on its natural abundance, large larval biomass, or feasibility of mass rearing. This shift in emphasis would better align the introduction with the study’s core focus on bioprospecting and make the organism selection appear more deliberate and scientifically justified.

2. The section describing the models for temperature and pH effects (Sections 2.5.2 and 2.5.3) is comprehensive, but the large number of equations (CTMI, BM, WMM, AYM, SRM, BPM, MeM1, MeM2) may overwhelm readers who are less familiar with mathematical modelling. To improve accessibility, I suggest that the authors provide a brief, plain‑language explanation of the biochemical meaning of each model. The full derivations could be moved to supplementary material, with only the key equations and parameter definitions retained in the main text.

3. In Section 3.1, the authors conclude that “R. palmarum larvae constitute a relevant biological source of glycosidases with competitive catalytic properties.” Whether this statement is fully justified by the data presented in Table 1. The final specific activity (28.67 U mg⁻¹) and the overall purification yield (2.22%) are relatively modest. Therefore, describing the enzyme as having “competitive catalytic properties” based solely on these purification metrics appears somewhat overstated. To strengthen their argument, the authors should bring forward additional evidence to highlight the unique advantages of this enzyme. Alternatively, if no clear superiority over existing enzymes can be demonstrated, I would suggest revising the sentence to a more measured and accurate tone.

4. The conclusions highlight the enzyme’s activity at moderately acidic pH, which is indeed promising for industrial uses. However, the current discussion remains somewhat general when it mentions applications such as lactose hydrolysis in fermented dairy products (e.g., yoghurt, pH 4.0–4.5). To make this argument more compelling and quantitatively meaningful, I would encourage the authors to provide the actual relative activity (or residual activity) of the enzyme at pH 4.0 and pH 4.5, based on their experimental pH‑activity profile.

5. Throughout the manuscript, abbreviations such as oNPG and pNPG are used; however, the full names and their definitions should appear only at their first occurrence, and thereafter the abbreviations alone should be used consistently. In addition, the use of significant figures deserves attention. For the same parameter – for example, the optimal temperature Tₒₚₜ or the Michaelis constant Kₘ – the reported values from different models or replicates should be expressed with a consistent number of decimal places or significant digits. Currently, some values are given with one decimal, others with two, which may create an impression of varying precision.

Reviewer #2: The manuscript titled “Kinetic and physicochemical modeling of β-galactosidase from Rhynchophorus palmarum larvae” presents a physicochemical and kinetic characterization of β-galactosidase from Rhynchophorus palmarum larvae using empirical and mechanistic modeling approaches. The study is methodologically sound, and the application of multiple models to determine optimal conditions and kinetic parameters represents a valuable contribution to the field. The enzyme shows promising characteristics for industrial applications. However, at the presented state the manuscript needs revision. The main comments and recommendations are listed below.

1. The introduction establishes the general importance of β-galactosidases and mentions that insect-derived enzymes have received little attention. However, the specific research gap being addressed is not clearly articulated. The authors state that previous studies investigated pH and temperature effects on this enzyme but did not incorporate modeling approaches. This is mentioned but not developed into a clear hypothesis or research objective.

2. This study appears to use the same enzyme source and purification method as Yapi et al. (2007), yet the authors report notably different values for several parameters. While some differences are attributed to nonlinear regression towards graphical methods" this explanation is insufficient.

3. The authors use both empirical and mechanistic models for temperature and pH effects. Explain how the parameter estimates from different models should be interpreted and why the mechanistic estimates are considered more reliable

4. The pK values for the enzyme-substrate complex are interpreted as corresponding to the nucleophile and proton donor, respectively. However, this interpretation requires stronger support with relevant references.

5. The study uses only oNPG as substrate. While this is a standard synthetic substrate for β-galactosidase assays, the absence of data with natural substrates (lactose) or other galactosides limits the practical relevance. Justify or add limitations.

6. The manuscript focuses on optimal temperature but provides limited information on thermostability

7. The discussion on enzyme stability and catalytic activity should be strengthen by expanded comparative analysis with the recent reported data. For example, https://doi.org/10.1016/j.ijbiomac.2026.152774

8. Conclusions should be better supported by the key data obtained. Clearly state limitations and future perspectives.

9. The text should be carefully checked for typos and grammatical errors.

Reviewer #3: The manuscript addresses a relevant topic and is generally well organised. However, I recommend a minor revision before it can be considered suitable for publication.

The manuscript requires careful proofreading and language editing to improve grammatical accuracy, clarity, and academic fluency. In addition, there are several typographical errors, punctuation inconsistencies, and minor formatting issues throughout the text that should be corrected. The authors should also ensure consistency in terminology, abbreviations, and sentence structure across the manuscript.

Overall, no major methodological or conceptual revisions are required. However, the manuscript should be revised carefully in terms of language, typographical accuracy, and presentation quality.

Recommendation: Minor Revision

**********

6. PLOS authors have the option to publish the peer review history of their article (what does this mean?). If published, this will include your full peer review and any attached files.

If you choose “no”, your identity will remain anonymous but your review may still be made public.

**Do you want your identity to be public for this peer review?**  For information about this choice, including consent withdrawal, please see our Privacy Policy.

Reviewer #1: No

Reviewer #2: No

Reviewer #3: No

**********

[NOTE: If reviewer comments were submitted as an attachment file, they will be attached to this email and accessible via the submission site. Please log into your account, locate the manuscript record, and check for the action link "View Attachments". If this link does not appear, there are no attachment files.]

To ensure your figures meet our technical requirements, please review our figure guidelines: https://journals.plos.org/plosone/s/figures

You may also use PLOS’s free figure tool, NAAS, to help you prepare publication quality figures: https://journals.plos.org/plosone/s/figures#loc-tools-for-figure-preparation.

NAAS will assess whether your figures meet our technical requirements by comparing each figure against our figure specifications.

# Author comment: Author response to Decision Letter 1

**DOI:** 10.1371/journal.pone.0354469.r002

7 Jul 2026

Reviewer #1

1-The introduction devotes too much attention to the agricultural damage caused by Rhynchophorus palmarum and insufficient emphasis on its potential as a source of digestive enzymes.

Response:

We thank the reviewer for this valuable suggestion. The introduction has been revised to reduce the emphasis on the agricultural impact of Rhynchophorus palmarum and its role as a vector of red ring disease. Additional information has been incorporated regarding the digestive physiology of the larvae, their abundance, their large larval biomass, and their potential as a promising biological source of glycosidases. This modification strengthens the rationale for selecting this organism in the context of enzyme bioprospecting.

2-The large number of mathematical models may overwhelm readers.

Response:

We appreciate this comment. To improve readability, we have added a brief biochemical interpretation of each model in plain language. In addition, we have simplified the presentation of the modelling framework and reduced unnecessary mathematical details in the main text while retaining essential equations and parameter definitions.

3-The statement describing the enzyme as having "competitive catalytic properties" appears overstated.

Response:

We agree with the reviewer. The sentence has been revised to adopt a more balanced interpretation of the results. The expression “competitive catalytic properties” has been replaced by “promising catalytic properties” to better reflect the experimental evidence.

4-Provide actual activity values at pH 4.0 and 4.5.

Response:

Thank you for this suggestion. The discussion section has been expanded to include the relative activity values observed at pH 4.0 and pH 4.5 (19.36 and 29.7 U mg ⁻¹, respectively).

5-Improve consistency of abbreviations and significant figures.

Response:

The manuscript has been thoroughly revised to ensure consistency in abbreviations, nomenclature, and significant figures throughout the text, tables, and figures.

Reviewer #2

1-The specific research gap is not clearly articulated.

Response:

We thank the reviewer for this important observation. The introduction has been revised to clearly identify the existing knowledge gap. Previous studies on β-galactosidase from Rhynchophorus palmarum mainly focused on purification and basic biochemical characterization. However, no comprehensive physicochemical modelling based on empirical and mechanistic approaches has been reported. The objective and originality of the present study have been clarified accordingly.

2-Differences with Yapi et al. (2007) require further explanation.

Response:

We agree with the reviewer. The Discussion has been expanded to provide a more detailed comparison with Yapi et al. (2007). We now discuss the influence of parameter estimation methods, particularly nonlinear regression versus graphical linearization, on the resulting kinetic and physicochemical parameters. For some kinetic parameters as Km, we can say that the difference could be explained primarily by the fact that we did not use exactly the same test conditions, particularly with regard to substrate concentrations. Furthermore, when using insect larvae as an enzyme source, the Km may also depend on the age (maturity) of the larvae and on the organ or tissue (midgut, salivary gland, liver, etc.).

3-Explain why mechanistic models are considered more reliable.

Response:

We thank the reviewer for this insightful comment. We have revised the discussion section to clarify the interpretation of parameter estimates obtained from empirical and mechanistic models. Empirical models are primarily mathematical descriptions designed to reproduce the experimental bell-shaped profiles of enzyme activity as a function of temperature or pH. Their parameters (Tmin, Tmax, pHmin, pHmax,…) are useful for precisely identifying optimal conditions and defining the range of activity, but they generally have no direct physicochemical significance. In contrast, mechanistic models are derived from enzyme kinetics and thermodynamic principles. Their parameters have explicit biochemical interpretations. Regarding temperature dependence, the estimated activation energy (Ea) reflects the energy barrier of the catalytic process, while the deactivation energy (Ed) quantifies the thermal inactivation of the enzyme. Regarding pH dependence, the estimated values of pKES1 and pKES2 correspond to the apparent pK values of the ionizable catalytic groups within the enzyme-substrate complex, thereby providing information on the protonation states required for catalysis. Although empirical models sometimes yielded a better statistical fit, mechanistic models were deemed more reliable for interpreting the parameters, as they are constrained by the underlying physicochemical mechanisms that govern enzymatic catalysis. Consequently, their estimated parameters are biologically relevant and can be directly compared to values reported for other enzymes, whereas empirical parameters primarily describe the shape of the curve without providing mechanistic information.

4-Interpretation of pKa values requires stronger support.

Response:

We agree that the assignment of apparent pKES values to the catalytic nucleophile and the acid/base proton donor must be supported by references. We have therefore revised the discussion section to clarify that these attributions are based on the well-established catalytic mechanism of retention glycoside hydrolases, particularly β-galactosidases. In these enzymes, the catalytic process involves two essential carboxylate residues: one acting as the catalytic nucleophile, which must remain deprotonated, and the other as a general acid/base catalyst (proton donor), which must remain protonated during the cleavage of the glycosidic bond. Consequently, the lowest apparent pKES value is assigned to the nucleophile, while the highest value is assigned to the proton donor. We have included the relevant reference, notably Davies and Henrissat (1995).

5-Only oNPG was used as substrate.

Response:

We thank the reviewer for this valuable comment. We agree that although oNPG is the standard chromogenic substrate widely used for the characterization of β-galactosidase due to its high sensitivity and convenient spectrophotometric detection, it does not fully represent the enzyme’s catalytic behavior toward its natural substrate, lactose. In the revised manuscript, we have explicitly acknowledged this limitation in the discussion (conclusion section). We also emphasize that future work will focus on validating kinetic and mechanistic models using lactose and other natural galactosides, as well as on evaluating the enzyme under conditions relevant to the industrial hydrolysis of lactose. These additions clarify the scope of the present work while outlining important directions for future research.

6-The manuscript focuses on optimal temperature but provides limited information on thermostability.

Response:

We fully agree with the reviewer’s pertinent comment. In this study, we focused exclusively on the short-term effect of temperature on enzymatic activity (thermotolerance) in order to optimize operational parameters through empirical and mechanistic modeling. Since we did not conduct time-dependent thermal inactivation assays (pre-incubation experiments), we cannot address the concept of “thermostability” in the strict kinetic sense. To avoid any misunderstanding and ensure absolute scientific rigor, we have carefully revised the manuscript. All instances of the terms “thermostability” and “thermal stability” have been replaced with “thermotolerance” or “thermal tolerance.”

7-The discussion on enzyme stability and catalytic activity should be strengthen by expanded comparative analysis with the recent reported data. For example, https://doi.org/10.1016/j.ijbiomac.2026.152774

Response:

We sincerely thank the reviewer for bringing this very recent and relevant study to our attention (Morozova et al., 2026, Int. J. Biol. Macromol.). We have carefully read the suggested article and have expanded our comparative analysis accordingly.

In the revised version of the manuscript, we have expanded the comparative analysis of the catalytic behavior of our enzyme with that of the modified β-galactosidase from Bifidobacterium longum BIM B-813 D. We have demonstrated that, while the B. longum enzyme functions effectively up to 50 °C, the β-galactosidase from R. palmarum larvae exhibits an even higher optimal temperature (57 °C / 330.0 K), highlighting its apparent exceptional thermotolerance. We also noted the interesting similarity in their optimal pH profiles (pH 5.0). This comparison significantly strengthens the biotechnological positioning of our biocatalyst.

8-Conclusions should be better supported by the key data obtained. Clearly state limitations and future perspectives.

Response:

We have thoroughly rewritten the Conclusion section to better emphasize our quantitative findings while explicitly addressing the limitations and future directions of this research.

Specifically, we have:

(i) summarized the key modeling and operational data (Topt at 57 °C, pH = 5.0, WMM/AYM validation);

(ii) explicitly stated the limitation regarding the non-using of natural substrate (lactose);

(iii) outlined clear future perspectives, including enzyme purification, immobilization strategies, and industrial application trials (lactose hydrolysis).

9-The text should be carefully checked for typos and grammatical errors.

Response:

We appreciate this recommendation. The entire manuscript has been thoroughly reviewed and edited to correct typos, improve sentence structure, and ensure standard English scientific style throughout.

Reviewer #3

The manuscript requires careful proofreading and language editing to improve grammatical accuracy, clarity, and academic fluency. In addition, there are several typographical errors, punctuation inconsistencies, and minor formatting issues throughout the text that should be corrected. The authors should also ensure consistency in terminology, abbreviations, and sentence structure across the manuscript.

Response:

We greatly appreciate the reviewer’s careful evaluation and constructive suggestions. Following the reviewer’s recommendation, the manuscript underwent a comprehensive linguistic revision to improve grammatical accuracy, clarity, readability, and the fluidity of the writing style. Particular attention was paid to correcting typos, punctuation inconsistencies, formatting issues, and sentence structure. In addition, we carefully reviewed the manuscript to ensure consistency in terminology, abbreviations, symbols, units, significant figures, and writing style throughout all sections, tables, and figures. We are confident that these revisions have significantly improved the overall quality and presentation of the manuscript.

Attachment Submitted filename: Response to Reviewers.docx

# Editor report: Decision Letter 1

**DOI:** 10.1371/journal.pone.0354469.r003

**Authors:** Rafael Vazquez-Duhalt

9 Jul 2026

Kinetic and physicochemical modeling of β-galactosidase from *Rhynchophorus palmarum*  larvae

PONE-D-26-15402R1

Dear Dr. KAMBIRE,

We’re pleased to inform you that your manuscript has been judged scientifically suitable for publication and will be formally accepted for publication once it meets all outstanding technical requirements.

Within one week, you’ll receive an e-mail detailing the required amendments. When these have been addressed, you’ll receive a formal acceptance letter and your manuscript will be scheduled for publication.

An invoice will be generated when your article is formally accepted. Please note, if your institution has a publishing partnership with PLOS and your article meets the relevant criteria, all or part of your publication costs will be covered. Please make sure your user information is up-to-date by logging into Editorial Manager at Editorial Manager® and clicking the ‘Update My Information' link at the top of the page. For questions related to billing, please contact billing support.

If your institution or institutions have a press office, please notify them about your upcoming paper to help maximize its impact. If they’ll be preparing press materials, please inform our press team as soon as possible -- no later than 48 hours after receiving the formal acceptance. Your manuscript will remain under strict press embargo until 2 pm Eastern Time on the date of publication. For more information, please contact onepress@plos.org.

Kind regards,

Rafael Vazquez-Duhalt, Ph.D.

Academic Editor

PLOS One

Additional Editor Comments (optional):

All the reviewer's comments and suggestions have been correctly addressed.

Reviewers' comments:

# Editor report: Acceptance letter

**DOI:** 10.1371/journal.pone.0354469.r004

**Authors:** Rafael Vazquez-Duhalt

PONE-D-26-15402R1

PLOS One

Dear Dr. KAMBIRE,

I'm pleased to inform you that your manuscript has been deemed suitable for publication in PLOS One. Congratulations! Your manuscript is now being handed over to our production team.

At this stage, our production department will prepare your paper for publication. This includes ensuring the following:

* All references, tables, and figures are properly cited

* All relevant supporting information is included in the manuscript submission,

* There are no issues that prevent the paper from being properly typeset

You will receive further instructions from the production team, including instructions on how to review your proof when it is ready. Please keep in mind that we are working through a large volume of accepted articles, so please give us a few days to review your paper and let you know the next and final steps.

Lastly, if your institution or institutions have a press office, please let them know about your upcoming paper now to help maximize its impact. If they'll be preparing press materials, please inform our press team within the next 48 hours. Your manuscript will remain under strict press embargo until 2 pm Eastern Time on the date of publication. For more information, please contact onepress@plos.org.

You will receive an invoice from PLOS for your publication fee after your manuscript has reached the completed accept phase. If you receive an email requesting payment before acceptance or for any other service, this may be a phishing scheme. Learn how to identify phishing emails and protect your accounts at https://explore.plos.org/phishing.

If we can help with anything else, please email us at customercare@plos.org.

Thank you for submitting your work to PLOS ONE and supporting open access.

Kind regards,

PLOS ONE Editorial Office Staff

on behalf of

Dr. Rafael Vazquez-Duhalt

Academic Editor

PLOS One
